# Not a matter of quantity: quality of relationships and personal interests predict university students’ resilience to anxiety during CoViD-19.

**DOI:** 10.1007/s12144-021-02076-w

**Published:** 2021-07-26

**Authors:** Marta Nola, Cecilia Guiot, Stefano Damiani, Natascia Brondino, Roberta Milani, Pierluigi Politi

**Affiliations:** 1 Department of Brain and Behavioral Sciences, Unit of Psychiatry, University of Pavia, Via Bassi 21, 27100 Pavia, Italy; 2 Department of Brain and Behavioral Sciences, Unit of Applied Psychology, University of Pavia, Piazza Botta 11, 27100 Pavia, Italy

**Keywords:** University students, COVID-19, Anxiety, Relationships, Interests, Community resilience

## Abstract

During the CoViD-19 pandemic, University students may have suffered from increased anxiety due to interferences in their relationships and in academic requirements, as didactic activities have moved to distance learning systems. However, being surrounded by supportive relationships and being motivated to cultivate personal interests might have decreased anxiety. In this pilot study, we collected the responses of 174 students from Italian University merit colleges to an online questionnaire, investigating their perceived anxiety, the quality of surrounding relationships, whether they were cultivating any personal interests and whether they had spent the period of lockdown in college or at home. Regression analyses indicated that both quality of relationships and personal interests predicted low levels of anxiety (*p* < 0.001). However, simple slope analyses showed that personal interests were negatively related to anxiety only at medium and high quality of relationships (*p* < 0.001), while no association was found at low quality of relationships. No differences were found between students who stayed in college or at home. These results suggest that Universities should promote accessibility to relationships and cultivation of personal interests to protect students’ mental health during mass emergencies such as the current pandemic, in the perspective of improving community resilience.

## Introduction

The CoronaVirus Disease 2019 (CoViD-19) outbreak originated in China at the end of 2019; in the first months of 2020, it has rapidly spread to a global scale, leading the World Health Organization to declare it as a pandemic on March 11th (World Health Organization 2020). In many Countries, the enforcement of preventive measures to contain the pandemic included the institution of mass quarantine and lockdown, which were supposed to take a toll on the psychological well-being of the general population (Brooks, et al. 2020) in addition to the effects of the pandemic itself. According to the first studies in China (Liu, Zhang, Wong, Hyun & Hahm 2020; Qiu et al. 2020; Wang et al. 2020; Zhang & Ma 2020; Cao et al. 2020) anxiety, depression and stress affected significant proportions of the population during the emergency. Data have shown that young adults and individuals with high levels of education represent a population at higher risk for psychological distress during the pandemic (Qiu et al. 2020); this may suggest that University students may constitute a more fragile population and that they may require special attention and care (Cao et al. 2020; Talevi et al. 2020; Sahu 2020; Liu, Zhu, et al. 2020) – as also supported by what happened during epidemiological crises in the past years (Al-Rabiaah et al. 2020). In particular, students suffered a higher psychological impact compared with University staff during lockdown (Odriozola-González, Planchuelo-Gómez, Irurtia, & de Luis-García 2020; Marelli et al. 2020).

There is also a need for robust data concerning the impact of CoViD-19 in the European socio-cultural context (Giallonardo et al. 2020). Italy was one of the Countries to be most severely struck by the pandemic in the very first months of 2020, with the region of Lombardy being particularly affected: as of September 8th, the region has reached a total number of cases of 102,085 over a national count of 280,153, with the impressive number of 16,888 over 35,563 deaths (Italian Ministry of Health 2020). With the lockdown on March 9th (Government of Italy 2020), all the educational and didactic activities (including Universities) were suspended or turned into distance learning as a precautionary measure.

During the lockdown, University merit colleges and residence halls have applied strict rules in order to allow for a safe cohabitation between the students who chose to stay instead of returning to their homes. This led to the creation of peculiar closed environments, whereby groups of students could face the social and mental strains of the pandemic as a community, providing reciprocal company and support in a way that could be compared to a proper domestic atmosphere. Crucially, in a time when most individuals were constrained in isolation, the maintenance of educational and social activities through online platforms might have contributed to a sense of belonging by involving students who were living with their families.

On the other hand, the sudden shift of teaching activities to online platforms might have determined impairments in learning and exam performance, enhancing distress in University students (Cao et al. 2020; Sahu 2020). It could be expected that such changes in academic procedures could impact primarily on students who are required to maintain higher standards in their grades, such as students who need to retain scholarships or those who live in merit colleges, which set high requirements in academic achievements (Italian Ministry of Education, University and Research 2020). Academic performance and pressure to succeed are relevant sources of concern for students, displaying a positive correlation with levels of depression, anxiety and stress (Beiter et al. 2015).

According to previous studies, social support from family and friends may be a protective factor for students’ mental health, especially for what concerns depression (Alsubaie, Stain, Webster & Wadman 2019), academic stress (Glozah 2013) and quality of life (Alsubaie et al. 2020). The supportive role of peers is particularly relevant during the years of University, in parallel with the process of individualization from family (Alsubaie et al. 2020). Conversely, the experience of loneliness, which is highly prevalent in young adults during University years (Hawthorne 2008; Matthews et al., 2016; Lim, Eres & Vasan 2020), may constitute a risk factor for the development of common mental health conditions (Matthews rt al., 2016; Lim et al. 2020; Nuyen et al., 2020). This aspect appears to be exceptionally relevant in the context of social isolation during the CoViD-19 pandemic (Loades et al. 2020; Foundation MH 2020).

As the measures of quarantine and lockdown entail staying at home for an exceptionally prolonged period of time, some researchers have questioned the role that housing and home environment can play on mental health in this setting (Amerio et al. 2020). While building design strategies are surely relevant to this concern, we argue that the quality of housing is also significantly determined by the cohabitation with other individuals. A study by Cao et al. (2020) indicates that living with parents might have been a protective factor against the development of feelings of anxiety for students during the CoViD-19 crisis. Notably, the authors have also observed that social support was negatively correlated with anxiety in this population (Cao et al. 2020). However, to our knowledge, the quality of relationships in the environment where individuals spent lockdown has not been investigated so far.

Based on these premises, the perception of being surrounded by a supportive relational network can be tentatively considered as a useful resource to tackle the mental health challenges raised by the CoViD-19 pandemic. In order to deepen the knowledge on this topic, we conducted an explorative investigation on anxiety and perceived quality of relationships during the CoViD-19 crisis in a population of students belonging to University merit colleges in Italy. Specifically, we hypothesized that spending the period of lockdown in a context with a high quality of relationships would constitute a protective factor against feelings of anxiety. In addition, we also explored whether the perception of having several interests in one’s life during the pandemic could act as a potential protective factor itself. Moreover, we investigated whether this finding would be similar in students who remained in their college community with respect to those who returned to their family homes.

## Methods

### Participants

Students from University merit colleges were involved irrespectively of having spent the three-month period of lockdown from March to May 2020 in college or at home. All recruited students were similarly involved in educational activities provided by the colleges on online platforms. All participants provided informed consent upon enrolment. Criteria for inclusion were to be resident at a University merit college for the academic year 2019–2020. In total, we received 193 responses, of which 2 responses were excluded as the subjects declared to not belong to a University merit college and 17 responses were excluded as more than half of the items were not completed. The final sample included 174 responses.

### Procedure

Enrolment took place in the time frame from June 20th to July 20th, 2020. A questionnaire on Google Form was distributed online by contacting rectors of University merit colleges, who spread the questionnaire to their students by email. Participation to the study was recognized as a credit-awarding educational activity in colleges. Ethical approval was waived by the internal review board of the Department of Brain and Behavioral Sciences of the University of Pavia.

### Survey

Questions relative to demographic information concerned gender, age, place of residence, and socioeconomic status. As our research was a pilot study, in order to explore relevant domains in our sample we included in our survey items from validated instruments and scales (Spitzer, Kroenke, Williams, & Löwe 2006; Butcher, Graham, Tellegen, & Kaemmer 1989). The tool answered by the participants can be found in the Appendix.

### Data Analysis

Data were analyzed with SPSS version 26. All measures, manipulations and exclusions applied in the study are reported. Descriptive statistics were calculated for all variables. Pearson correlation coefficient *r* was used to assess correlation between variables (place of residence during lockdown, gender, socioeconomic status, feeling anxious, perceived quality of personal relationships and personal interests). For our variables of interest (perceived anxiety, perceived quality of personal relationships and personal interest, partial correlations after controlling for demographic variables were also assessed. A one-way ANOVA was performed comparing levels of anxiety according to place of residence during the lockdown. In order to best understand the relationship between our focus variables, we performed a three-step multiple linear regression analysis. Finally, we performed a simple slope analysis in order to understand whether the quality of relationships could act as a moderator with respect to the effect of personal interests on levels of anxiety. A two-tailed *p* < 0.05 was considered statistically significant.

## Results

Table 1 shows the descriptive statistics for the variables of interest in our study. Of 174 participants, 121 identified themselves as females, 49 as males, and 4 as other (transgender, intersexual, gender fluid or non-binary). The mean age of participants was 21.70 years (SD = 2.15). Among the participants, more than 90% belonged to a merit college situated in Lombardy, and 58.62% was in Lombardy when responding to the questionnaire; in particular, 57.5% spent the lockdown entirely at home, 10.9% were in college for less than one month, and 30.5% were in college between one and three months. Participants reported their socioeconomic status to be low (6.9%), intermediate (74.7%), or high (17.2%), while 1.1% of participants did not respond to this question.

**Table 1:** Descriptive variables and sample demographics

Variable	N^†^ (%)	Feelings of anxiety
Gender		
Male	49 (28.2%)	5.37 (2.970)
Female	121 (69.5%)	6.76 (2.754)
Other	4 (2.3%)	‡
Socioeconomic status		
Low	12 (6.9%)	8.17 (1.80)
Intermediate	130 (74.7%)	6.18 (2.888)
High	30 (17.2%)	6.60 (3.047)
Place of residence		
College (1–3 months)	53 (30.5%)	6.51 (2.757)
College (< 1 month)	19 (10.9%)	7.05 (2.656)
Home	100 (57.5%)	6.11 (3.038)
Full sample	174 (100%)	6.35 (2.90)

N^†^ = number; ^‡^ the analysis could not be carried out for this group due to its small number. Values for feelings of anxiety, quality of relationships and personal interests are reported as mean (standard deviation)

Correlations between our control and focus variables are reported in Table 2. Significant correlations were found between feeling anxious and both quality of relationships (*r* = −0.43, *p* < 0.001) and personal interests (*r* = −0.45, *p* < 0.001), which remained significant even when we controlled for place of residence during lockdown, gender, and socioeconomic status.

**Table 2:** Correlation analysis including control and focus variables

		1	2	3	4	5	6
1. Gender	*r*						
	*p*^‡^	–					
	N^§^						
2. Socioeconomic status	*r*	−0.093					
	*p*	0.226	–				
	N	172					
3. Place of residence	*r*	0.014	−0.047				
	*p*	0.858	0.543	–			
	N	172	170				
4. Feelings of anxiety	*r*	0.163	−0.182	0.066			
	*p*	< 0.032	0.017	0.387	–		
	N	174	172	172			
5. Quality of relationships	*r*	−0.151	0.291	0.081	−0.434 (−0.407^¶^)		
	*p*	0.048	< 0.001	0.291	< 0.001	–	
	N	173	171	171	173		
6. Personal interests	*r*	−0.064	0.245	−0.082	−0.447 (−0.413^¶^)	0.513 (0.509^¶^)	
	*p*	0.402	0.001	0.283	< 0.001	< 0.001	–
	N	174	172	172	174	173	

*r*^†^ Pearson correlation coefficient; *p*^‡^ *P* value; N^§^ number of responses; ^¶^ partial correlations after controlling for control variables (gender, socioeconomic status, place of residence)

In order to assess whether levels of anxiety would differ between participants who spent the lockdown with their family and those who remained in their college community, we performed a one-way ANOVA. The difference in levels of anxiety between participants who stayed at home (M = 6.11, SD = 3.04), who stayed in college <1 month (M = 7.05, SD = 2.66) and those who stayed in college >1 month (M = 6.51, SD = 2.76) was not significant (F = 0.97, *p* = 0.382).

We then performed a multiple linear regression analysis as shown in Table 3. We considered feeling anxious as the dependent variable. At Step 1 we entered control variables (place of residence during lockdown, gender, and socioeconomic status); at Step 2 we entered focus variables (quality of personal relationships and personal interests). Step 1 was significant (*R*^*2*^ = 0.06, *p* = 0.02), with socioeconomic status being the only significant predictor. The addition of focus variables at Step 2 significantly improved the amount of variance explained (*ΔR*^*2*^ = 0.21, *p* < 0.001); crucially, both quality of relationships and personal interests resulted as significant predictors. We then added Step 3 in the regression in order to investigate whether the quality of relationships had a moderating effect in the relation between personal interests and feeling anxious. The findings indicated that the addition of the interaction term personal interest * quality of relationships explained a unique 5% in feeling anxious (*ΔR*^*2*^ = 0.05, *p* < 0.001). Finally, following the procedure by Cohen et al. (2003), we probed the effect of personal interests on feeling anxious at low, medium and high levels of quality of relationships. Simple slope analyses indicated that personal interests were negatively related to feeling anxious only at medium (*b* = −0.33, *p* < 0.001) and high (*b* = −0.59, *p* < 0.001) levels of quality of relationships (at exactly +1 SD above the mean). No relations between personal interests and feeling anxious were found for low levels of quality of relationships.

**Table 3:** Multiple linear regression analysis with feelings of anxiety as dependent variable

Variables	Step 1			Step 2			Step 3		
	*R*^*2*†^	*β*^‡^	*p*^§^	*ΔR*^*2*^	*β*	*p*	*ΔR*^*2*^	*β*	*p*
Control variables	0.056		0.02						
Gender		0.137	0.073		0.095	0.165		1.634	0.104
Socioeconomic status		−0.163	0.034		−0.019	0.791		−0.100	0.446
Place of residence		0.066	0.386		0.068	0.318		0.060	0.680
Focus variables				0.21		< 0.001			
Quality of relationships		–	–		−0.272	0.001		−0.461	< 0.001
Personal interests		–	–		−0.280	0.001		−0.333	< 0.001
Interaction term							0.051		< 0.001
Personal interests*quality of relationships		–	–		–	–		−0.110	< 0.001

*R*^*2*†^ coefficient of determination; *β*^‡^ standardized beta coefficient; *p*^§^ P value

## Discussion

Current research on the effects of the CoViD-19 pandemic and lockdown on mental wellbeing has shown that University students are at particular risk for anxiety (Wang et al. 2020; Cao et al. 2020; Talevi et al. 2020, Wang & Zhao 2020; Mei, Yu, He & Li 2011). In particular, the female gender was generally associated to higher levels of anxiety (Liu, Zhang, et al. 2020; Qiu et al. 2020; Wang et al. 2020). In our correlation analysis, female participants do indeed show a tendency to report higher feelings of anxiety; however, in regression analysis, this observation is not significant, despite being close to the threshold (*p* = 0.073). Moreover, in our study, participants with a lower socioeconomic status have reported higher feelings of anxiety compared to those who considered their socioeconomic status to be intermediate or high. Similarly, other studies have shown that occupation (Talevi et al. 2020) and family income stability (Cao et al. 2020) influence psychological distress. In particular, students’ anxiety appears to be related to worries about the economic consequences of the pandemic (Cao et al. 2020). However, this finding was significant only at Step 1 of regression analysis, and lost significance when focus variables were added.

Cao et al. (2020) have observed that living with parents seemed to be a protective factor against anxiety in students. In our study, instead, no differences were found between students who spent the period of lockdown with their families and those who stayed in their college community with peers. A possible interpretation of this finding could be that colleges provide comprehensive services and a stable relational network to students, thus being similar to a familiar, domestic environment. To these regards, it should be noted that students in our sample were allowed to choose whether to stay in college or to return to one’s family as the lockdown measures were instituted; however, the decision of each individual could have been made on the basis of a variety of personal and contextual factors, only in part pertaining to one’s perceived best interest.

This latter finding might be better understood considering the relation between feelings of anxiety and the perceived quality of relationships at the place where participants spent the period of lockdown. According to our results, a high quality of relationships would be related to lower levels of anxiety. Other studies have indeed shown that social support might be a protective factor against anxiety during the pandemic (Cao et al. 2020) and social support is known to be associated with better mental health in University students in general (Alsubaie et al. 2020; Glozah 2013). Indeed, close contact with nurturing relationships does not only provide access to care and support, fostering a sense of safety, but it also allows the individual to actively take care of others, promoting a sense of self-efficacy and self-esteem – a domain of great importance when facing disruptive, potentially traumatic events (Filippini, Semeraro, & Vilei 2020). Taken altogether, these observations may suggest that a key factor for mitigating anxiety during a pandemic would be to spend the period of lockdown together with people one has a good relationship with, and not necessarily to be with one’s family or to have supporting relationships outside of one’s place of residence. However, more targeted investigations should be carried out to test this hypothesis.

An additional factor that we took into consideration was the perception of having interesting things in one’s life during the pandemic. The tendency towards learning and exploring new things with curiosity and autonomy is related to internal motivation, and it is a key component of self-determination (Ryan & Deci 2000). Intrinsic motivation is an important factor for promoting accomplishment and performance in academic and educational environments (Afzal, Ali, Khan, & Hamid 2010). In particular, in the population we studied, college students were encouraged to take part to online social and educational activities of their choice, which were only in part related to academic requirements and were not associated with formal evaluations and results; in addition, they also had the goal to provide students with cultural and amusing stimuli that could help in managing stress and anxiety during the lockdown. For these reasons, these activities may have potentially fostered empowering instances such as sense of self-efficacy, internal motivation and self-determination in students. Moreover, online social activities may have also promoted the feeling of being part of a relational network in a period of significant isolation. In our findings, personal interests appear to have a protective role against feelings of anxiety, but this effect is significant only for medium and high levels of quality of relationships, while participants who reported a low quality of relationships at one’s place of residence seem to have gained little benefit from cultivating personal interests. This result highlights the importance of relationships with respect to other means of dealing with anxiety during lockdown in our population of study.

In summary, our pilot investigation has shown perceived good quality of relationships at the place of residence – irrespectively of it being the college or one’s family home – and being encouraged to cultivate personal interests during the pandemic are associated with reduced anxiety in University merit college students. We speculate that University merit colleges may be helpful for students during periods of stress such as public health emergencies by fostering a psychological sense of community – a concept first introduced by Seymour Sarason (1974) as “the sense that one was part of a readily available, mutually supportive network of relationships upon which one could depend” (p. 1), which could be protective against isolation and anxiety. Sense of community is recognized as a factor contributing to community resilience (Cheshire, Esparcia, & Shucksmith 2015), since a feeling of belonging to one’s community promotes active involvement in a collective response to disruptive events and facilitates access to social support (Paton & Johnston 2001). We might thus expect that during a public health emergency, young adults would benefit from a sense of belonging to a resilient community perceived as both a nurturing relational environment and a safe, reliable institution, capable of managing risks and of promoting the adaptability of its members to adverse events.

In conclusion, our study suggests that among the preventive measures to be applied in the face of mental health risks for University students during emergencies – such as counselling and psychological support – it is of key importance to promote accessibility to relationships and cultivation of personal interests. Consistently with their exploratory formulation, our observations are limited, as we did not employ structured, validated tools in our investigation. Moreover, our analyses were carried out with a limited number of participants, most of whom were from the same region and thus lacked in heterogeneity. Additional information concerning housing and accommodation of participants could also have been important to contextualize our results. Furthermore, we did not compare the population under study with other groups of students and young adults, which could be of interest in order to evaluate whether colleges do indeed provide additional benefits during the years of University respective to other environments. We believe that further investigations should be carried out to assess the protective role of a psychological sense of community and of internal motivation in students in the setting of mass emergencies, and to identify which aspects may be adopted in the University setting to improve community resilience.

## Appendix

**Items of the survey translated in English.**
